# The Periodic Replacement of Adhesive Setae in Pad Lamellae of Climbing Lizards Is Driven by Patterns of Corneous Layer Growth

**DOI:** 10.3390/jdb11010003

**Published:** 2022-12-30

**Authors:** Lorenzo Alibardi

**Affiliations:** 1 Comparative Histolab Padova, 35100 Padova, Italy; lorenzo.alibardi@unibo.it; 2 Department of Biology, University of Bologna, 40126 Bologna, Italy

**Keywords:** lizard, digits, adhesive pads, autoradiography, immunohistochemistry, shedding

## Abstract

The adhesive digital pads in some gecko and anoline lizards are continuously utilized for movements on vertical surfaces that may determine wear and a decrease of adhesion efficiency. The pads are formed by lamellae bearing adhesive setae that are worn out following frequent usage and are replaced by new inner setae that maintain an efficient adhesion. Whether the extensive usage of adhesive setae determines a higher shedding frequency in the digital pads with respect to other body regions remains unknown. Setae replacement has been analyzed in embryos and adult lizards using autoradiography and 5BrdU-immunohistochemistry. The observation strongly suggests that during development and epidermal renewal in adult lamellae, there is a shifting of the outer setae toward the apex of the lamella. This movement is likely derived from the continuous addition of proteins in the beta- and alpha-layers sustaining the outer setae while the inner setae are forming. Ultrastructural and in situ hybridization studies indicate that the thin outer beta- and alpha-layers still contain mRNAs and ribosomes that may contribute to the continuous production of corneous beta proteins (CBPs) and keratins for the growth of the free margin at the apex of the lamella. This process determines the apical shifting and release of the old setae, while the new inner setae formed underneath becomes the new outer setae.

## 1. Introduction on Lizard Adhesive Pads and Their Growth

**Figure 1:**
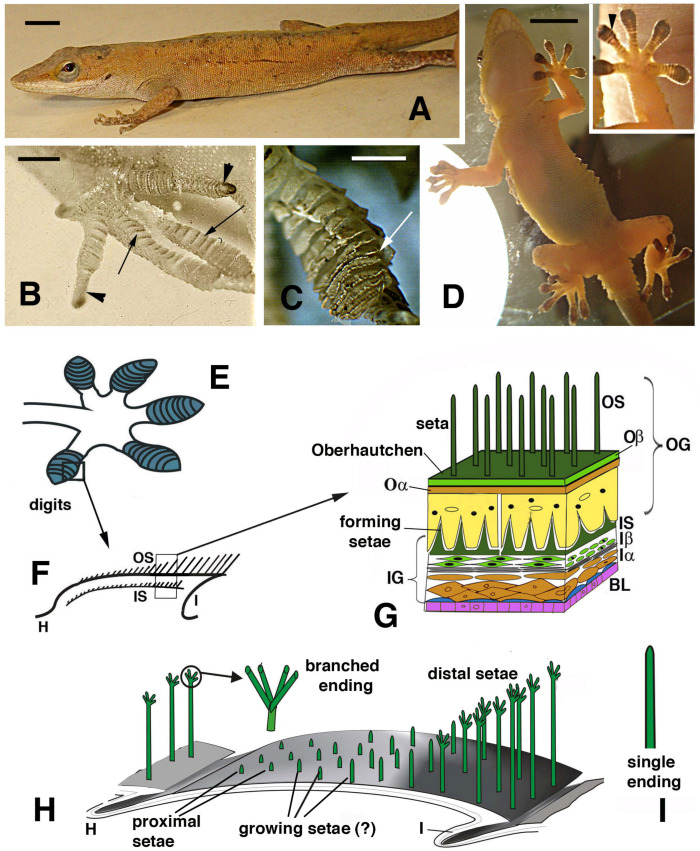
Lizards that can use digital adhesive pads (**A**–**D**) and schematic drawing of their histological and cellular structure (**E**–**H**). (**A**), *Anolis carolinensis*. Bar, 5 mm. (**B**), digits with developing lamellae (arrows) and claws (arrowheads) in an advanced embryo of *A. lineatopus*. Bar, 1 mm. (**C**), detail of overlapped lamellae (arrow) in adult *A. carolinensis*. Bar, 1 mm. (**D**), adult of *Tarentola mauritanica* climbing a glass wall as evidenced by a lamp-illuminated background. Bar, 10 mm. The inset details the adhesion of the hand pad lamellae (arrowhead) on the glass surface. (**E**), drawing of digits with pad lamellae (blue). (**F**), detail on the structure of a lamella sectioned longitudinally to show the two generations of setae: outer and the developing inner setae generation. (**G**), detail of the epidermal layers forming the outer and inner setae generations. (**H**), three-dimensional reconstruction of a pad lamella of geckos, evidencing the proximal–distal sequence of setae of different lengths (adhesion essentially occurs on the distal setae that do not overlap with the other lamellae). ? indicates that it is unknown whether setae growth on the outer setae takes place. The circle shows a magnification of the seta with its branching. (**I**), detail of an unbranched ending of a seta from *A. carolinensis* or *A. lineatopus*. **Legend**: BL, basal layer (in blue suprabasal cells); H, hinge (inter-scale) region; I, inner (ventral) scale/lamella surface; Iα, forming inner alpha-cells (differentiating); Iβ, forming inner beta-cells; IG, inner epidermal generation; IS, inner setae; Oα, outer alpha-layer; Oβ, outer beta-layer; OG, outer epidermal generation; OS, outer setae.

Among lizards, numerous species have evolved sticking devices on their digit endings and, in a few geckos, also at the tail end, that are indicated as adhesive pads and allow these lizards to adhere efficiently on surfaces of a variable nature [1,2,3,4,5,6,7]. While in most lizards the presence of digital claws and overlapping scales allow movements along rough surfaces such as tree bark, the amazing ability of some lizards to move onto vertical or even upside-down smooth surfaces of almost any material depends on the evolution of adhesive digital pads (Figure 1A–D) [1,2,3,4,5,6,7]. Aside from a few species of scincid lizards, most lizard species with adhesive pads belong to the gekkonic and gekkotan family [2,5,6,7], and some species also to iguanids such as the genus *Anolis* sp. [8,9,10,11,12]. In a few species of geckos, a number of tail scales also become adhesive, facilitating climbing in an invariably dense canopy of trees [13,14,15,16].

While the above reviews deal with the anatomical, ecological, behavioral and biomechanical functions of adhesive pads in geckos, the present review mainly summarizes recent cell biology studies on the adhesive setae of pad lamellae in some anoline lizards (*Anolis lineatopus* and *A. carolinensis*) and in various geckos (*Tarentola mauritanica, Hemidactylus turcicus (Woodworthia maculate*, Gray, 1845), *Phelsuma dubia, Hoplodactylus maculatus, Lygodactylus capensis, Gekko gecko, Sphaerodactylus argus*). Different morphological and microscopical methods were utilized on these species in addition to histology and electron microscopy, including autoradiography and immunohistochemistry [4]. The methodological details of these studies are indicated in the following sections on the development and renewal of adhesive pads and their setae (see below).

**Figure 2:**
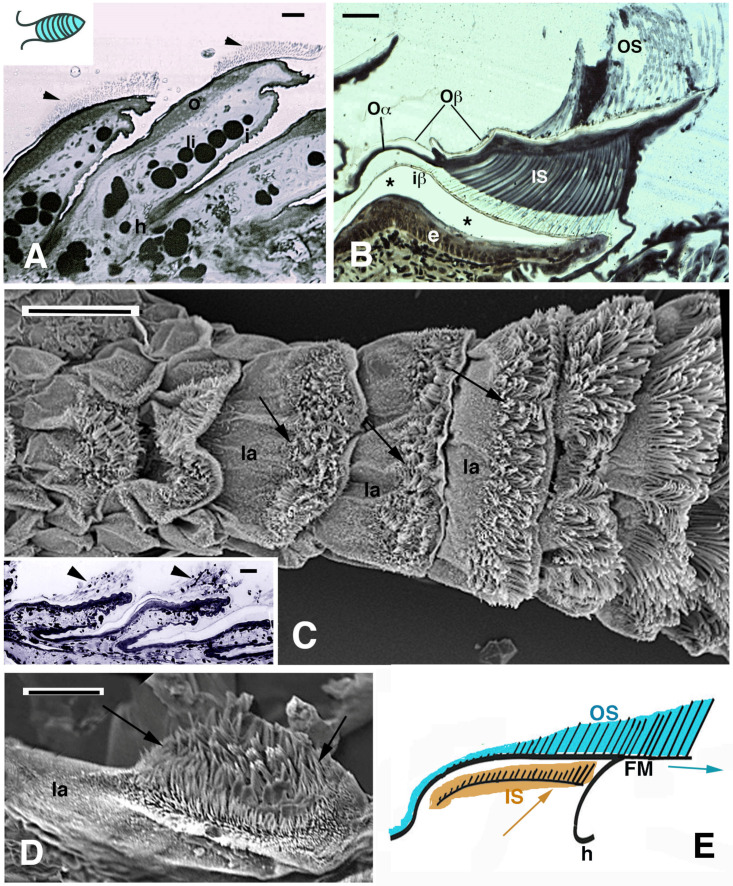
Histology (**A**,**B**) and SEM view (**C**–**E**) of digital lamellae in geckos. (**A**), longitudinal section of *Phelsuma dubia* showing a group of setae at the tip of the lamellae (arrowheads). Bar, 20 μm. (**B**), pad lamella of *Hemidactylus turcicus* featuring the outer and inner setae generations. Asterisks indicate an artifact detachment of the inner Oberhautchen-beta-layer from the epidermis. Bar, 20 μm. (**C**), SEM view of the ventral side of a digit in *Tarentola mauritanica*. Note the apical localization of the setae (arrows) in the lamellae. The longer setae are located in the distal lamellae. Bar, 200 μm. In the inset (Bar, 20 μm), the apical localization of setae (arrowheads) is evident in *Anolis carolinensis* lamellae. (**D**), apical collection of setae (arrows) in an isolated pad lamella of *H. turcicus* otherwise covered with spinulae. Bar, 50 μm. (**E**), schematic drawing of lamella with outer and inner generation of setae. The arrows indicate the presumed direction of shifting of the outer and inner setae. **Legend**: e, epidermis; FM, free margin (apical part of the Oberhautchen-beta-layer sustaining the apical outer setae); h, hinge (inter-scale/lamella) region; i, inner lamella surface; iβ, inner Oberhautchen-beta-layer; IS, inner setae generations; la, pad lamella; li, lipid droplets (cells); o, outer lamella surface; Oα, outer alpha-layer; Oβ, outer Oberhautchen-beta-layer; OS, outer setae generation.

Within a digital pad, the scales undergo an outstanding transformation of the external layer of the epidermis, namely, the Oberhautchen, which forms long bristles or setae that can reach over 100 μm in length (Figure 1G,H). Setae originated during the renewal phase of the shedding cycle of lizards, through a molding action of the clear layer of the outer epidermal generation on the Oberhautchen of the inner epidermal generation (Figure 1E–G) [4,17,18,19,20]. Setae terminate with a single spatula ending, as in *Anolis* sp., while in geckos, setae branch distally into a number of thinner branches that eventually terminate into spatulae (Figure 1H). Moreover, setae tend to concentrate from the distal half of a lamella to the tip where they are exposed on the surface and reach their maximum length (Figure 2A–E). The latter organization in the overlapped lamellae of pads allows the setae to remain exposed to the substrate, where they can stick. 

**Figure 3:**
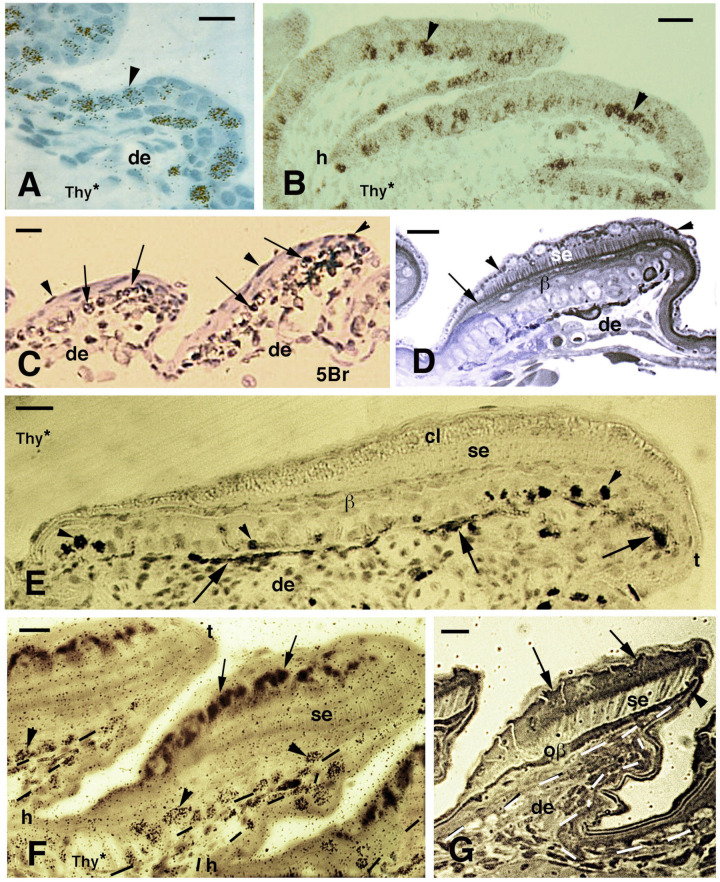
Histology of developing pad lamellae in *Anolis lineatopus* after tritiated thymidine autoradiography ((Thy*), (**A**,**B**,**E**,**F**)) or 5BrdU immunolabeling ((5Br), (**C**)). Toluidine blue and low-stained sections. Bars, 10 μm in all images. (**A**), initial formation of a lamella 4 h post-injection (arrowhead on labeled nuclei). (**B**), elongating lamellae 2 days post-injection (arrowheads on some labeled cells). (**C**), labeled nuclei in the basal (arrows) and periderm (arrowheads) at 4 days post-injection. (**D**), stained section showing the forming Oberhautchen (arrow) and the initial setae underneath the granular (clear) layer (arrowheads). (**E**), elongating lamellae with labeled cells (arrowheads) in the basal layer 2 days post-injection. Arrows indicate melanophores. (**F**), lamellae with developed setae 2 days post-injection of tritiated thymidine with labeled cells (arrowheads) visible in the basal layer (tangentially cut). Dashes outline the epidermis. Large granules (arrows) are present in the clear layer. (**G**), well-developed lamella (late embryo) evidencing the formation of the free margin (arrowhead) at the tip. Arrows indicate cells of the corneous clear layer. Dashes outline the epidermis. **Legend**: β, beta-layer/cells; cl, clear (granulated) layer; de, dermis; h, forming hinge (inter-scale) region; oβ, Oberhautchen-beta-layer; se, setae; t, lamella tip.

**Figure 4:**
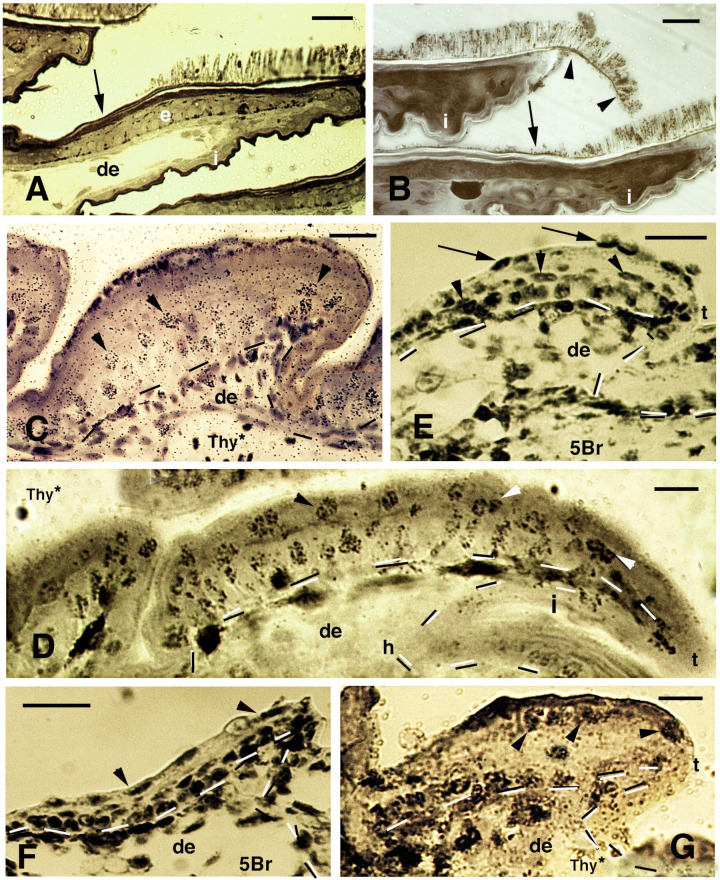
Histology of pads (**A**,**B**), sections after thymidine autoradiography ((Thy*), (**C**,**D**,**G**)) and after 5BrdU-immunohistochemistry ((5Br), (**E**,**F**)) in embryos of *A. lineatopus*. (**A**), pad with setae in the digit of a hatchling *A. lineatopus*. Setae are absent in the proximal part of the lamella (arrow) that is covered from the overlying lamella. Bar, 20 μm. (**B**), downfolded flexible free margin with distal setae (arrowheads) in the lamella of adult *A. carolinensis*. The arrow indicates lack of setae. Bar, 10 μm. (**C**), developing lamella of *A. lineatopus* 4 days post-injection showing basal and suprabasal (arrowheads) labeled cells. Bar, 20 μm. (**D**), elongating lamella (early stage) showing numerous labeled cells in most layers at 6–7 days post-injection, especially in the outer surface. Arrowheads indicate labeling in cells of the forming Oberhautchen. Bar, 10 μm. (**E**), short lamella located at the base (proximal part) of a digit, showing numerous labeled nuclei in the basal, suprabasal (arrowheads) and periderm (arrows) layers at 6–7 days post-injection. Bar, 20 μm. (**F**), other elongating lamella with most epidermal cells labeled at 4 days post-injection, including the periderm (arrowheads). Bar, 20 μm. (**G**), short proximal lamella with labeled cells in the basal layer and in clear cells (arrowheads) at 4 days post-injection. Bar, 10 μm. **Legend**: de, dermis; e, epidermis; i, inner surface of the lamella; t, tip of the lamella. Dashes outline the epidermis.

**Figure 5:**
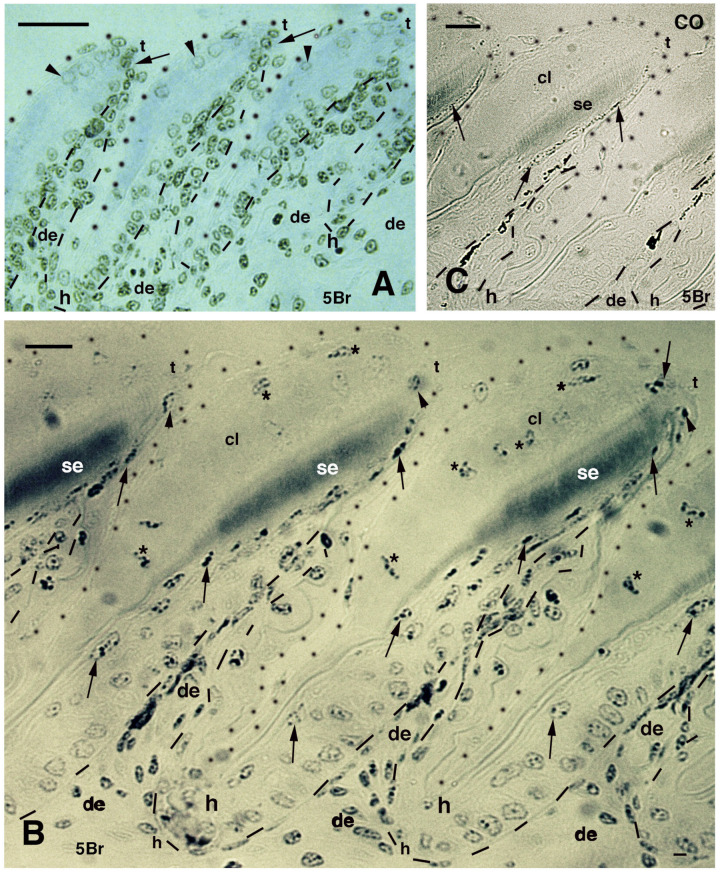
Immunolabeled pad lamellae of *A. lineatopus* at 8 days post-injection of 5BrdU (5Br). (**A**), early developing lamella with most labeled nuclei in the epidermis and dermis. Arrows indicate the relatively higher labeled nuclei by the tip of lamellae. Arrowheads point to labeled clear cell nuclei. Bar, 20 μm. (**B**), detail of labeled cells in the forming beta-layer (arrows pointing to flat nuclei). At the tip of the lamellae, labeled Oberhautchen cells are present (arrowheads). Asterisks indicate labeled nuclei of clear cells. Bar, 10 μm. (**C**), a control section does not show any labeled nuclei aside from finer grains of pigments (arrows). Bar, 10 μm. **Legend**: cl, clear layer; de, dermis; h, hinge (inter-scale) region; se, setae; t, tip (of the pad lamella). Dashes outline the epidermis. Dots outline most of the closely lined pad lamellae.

The formation of long setae creates a space issue in the lamellae since the outer growth of the inner setae needs space to elongate underneath the outer corneous layers (Figure 1F,G and Figure 2B). In order to cope with the extensive volumetric increase of the shedding complex forming underneath the outer generation of setae, the latter are not only lifted outward but also shifted toward the tip of the lamellae where they still continue their adhesive capacity until they are shed. This loss leaves space to the new (inner) lamellae that now become exposed over their overlapping lamellae to continue for a free adhesion. A histological study on the sub-digital lamellae of *Anolis carolinensis* provided two possible explanations for the process of setae replacement: (1) distal migration of differentiating cells that elongate the lamellae or (2) the regression of the beta- and alpha-layers and of the lamella dermis [17]. While the first explanation indicates the growth of scales, the latter process would determine a decrement of the scale size, although this would correspond to an increment of the length of the free margin. We are in favor of the first process indicating that an apical shifting is working in the enlarging lamella, due to the addition or the deformation (stretching) of new alpha/beta-cells that move distally during the outer epidermal generation. This process has been indicated through autoradiographic and in situ hybridization studies [21,22]. The lamellae of adult lizards that are still growing (*Anolis carolinensis* and *Hemidactylus turcicus*) were analyzed after thymidine, histidine and proline tritiated administration through autoradiography or by 5BrdU-immunohistochemistry after injection of 5BrdU. The details of these methods are reported in specific reported publications. Briefly, radioactive thymidine was injected into lizards and digit tissues were fixed at progressive periods after injection (4 h, 2 days, 4 days, 6–8 days, 12–13 days). The sections were covered with nuclear photographic emulsion (ILFORD), kept in the dark for 1–2 months, developed and fixed for autoradiography. Other lizards were injected with radioactive amino acids (histidine or proline) for the study of protein synthesis localization in specific epidermal layers of the adhesive pads at successive periods (4 h and 2 days post-injection). Autoradiographic sections were observed unstained or slightly stained with 1% toluidine blue. Other lizards were instead injected with the tag for DNA-synthesis 5BrdU at progressive post-injection periods, as above, in order to detect the localization of 5BrdU-incorporating (dividing) cells within the pads in successive periods. Antibodies against 5BrdU were utilized to detect the labeled nuclei. These studies have indicated possible cell movement toward the apex of pad lamellae as they grow. The study on developing embryos of the iguanid lizard *Anolis lineatopus*, using 5BrdU or tritiated thymidine, showed a likely shifting of cells still metabolically active in the alpha-layer, and even in the beta-layer, of the outer setae toward the apex of scales (Figure 3, Figure 4 and Figure 5, see the following descriptions).

A further indication that cells still alive may participate in an active movement of the corneous beta-layer sustaining the outer setae derives from the ultrastructural observation of the corneous layer sustaining the outer setae in *A.carolinensis*, *Gekko gecko* and *Tarentola mauritanica*. Details of the TEM study showing that the beta-layer of the outer scale contains still viable cells will be discussed later. The studies so far conducted have shown that differentiating beta-cells, and also likely alpha-cells produced in the basal layer, were not only moving vertically in the epidermis but also toward the scale tip, especially in the elongated and overlapping scales of the tail and limbs.

## 2. Development of Pad Lamellae (Scansors) in Lizards

The studies on the embryonic development of pads or entire scansors are very scarce and limited to few species, and the referring embryonic stages are hardly comparable. In general, pads are formed in the last third of embryonic development when other scales are also formed. In the gecko *Ptyodactylus guttatus* at the indicated stage L [23], the embryo features a large head with a developed eye and possesses developed limbs, digits, and tail but the skin is still smooth. On the ventral surface at the tip of the digits, the skin is still linear with a bilayered epidermis comprised of a periderm and a basal layer, like the epidermis formed over most of the body. In the following stage, indicated as Q by the first author that described this species, two circular swellings of the skin at the tip of the fingers indicate the future areas of formation of the pads (see also [24]). The skin is composed of a flat epidermis with some mesenchymal condensation underneath the swellings. At the successive stage S [see 24], the embryo has grown considerably and the digital swellings surface forms folds that move deeper and deeper into the dermis. It is from these skin folds that pads are originated, and they become evident at stage V (see [24]) when most of the body is also scaled and shows epidermal stratification with initial keratinization [24].

In the gecko *Tarentola annularis* and *T. mauritanica*, the sequence of formation of pads derives from an initial flat surface at stages 32–33 where digits are separated but the ventral digital surface remains smooth [25,26]. The skin is comprised of a bilayered epidermis but no dermal condensations are observed underneath the basement membrane. A series of skin folds is generated in the ventral aspect of digital tips at stages 34–35, and they become deeper in the following stages 36–37 when the scales elongate into lamellae that are clearly visible but initially poorly overlapped [25]. At stage 38, the epidermis stratifies while the lamellae elongate, while setae are formed at stage 39 after the differentiation of an Oberhautchen and beta-layer, some days before hatching. In another species, the gecko *Gecko japonicus*, pads even develop at later stages of embryogenesis, indicated as stages 38–39 when setae are not yet forming [27]. The latter form after stage 40 and are well seen at stage 42, a few days before hatching.

In the Jamaican iguanid lizard, *Anolis lineatopus*, adhesive pads are generated from embryonic stage 36. At this stage, the embryo is scaling over most of the body and the skin is gaining pigmentation while the tail is extended. On the ventral region of the apical digits, the formation of slanted scales become visible at stage 36 and they elongate and become overlapped at the successive stage 37, when the setae also initiate their formation [28,29] (Figure 3A–D). The autoradiographic and 5BrdU-immunohistochemical study on *A. lineatopus* reveals that an intense cell proliferation takes place in the epidermis, especially in the forming outer (dorsal) side of lamellae [28,29] (Figure 3). Labeled cells remain in the basal layer of scales at 4 h and 1-day post-administration of tritiated thymidine or 5BrdU (Figure 3A–C). The length of the forming setae grows in the outer side of the lamella while the epidermis produces fusiform cells of the beta-layer and the Oberhautchen forms setae that grow inside the cytoplasm of clear cells that contain numerous granules (Figure 3D). At 2 days post-injection, labeled cells still remain mainly in the basal layer in the early or later stages of lamella morphogenesis, with no differentiating cells labeled (Figure 3E,F). At stage 37, the beta-layer become compacted and forms a dense lamina beneath the first generation of setae that extends as far as the tip of the forming lamella, giving rise to the free margin (Figure 3G). At hatching, the free margin supports the longest group of setae of the adhesive lamellae (Figure 4A), while the free margin moves forward in adult lamellae until it is shed (Figure 4B). As a result of this apical growth of the setae, they always remain exposed to the surface and therefore exert their functionality.

During the elongation of the pad lamellae at stage 36, labeled nuclei with tritiated thymidine or 5BrdU are seen in suprabasal layers, especially by their elongating tips, 4 days after injection [28,29] (Figure 4C,D). Labeled keratinocytes in the differentiating clear and Oberhautchen cells are observed at 6–7 days also by the lamella tip (Figure 4F,G). Finally, at 8 and 13 days post-injection, numerous cells are labeled in most epidermal layers if the injection is made at the early stages (34–35) of pad formation (Figure 5). When injections are made at mid–late stages of embryonic development (36–37), labeled cells are seen in the large cells of the clear layer and in the flat cells of the Oberhautchen and beta-layers, especially by the tip of the pad lamellae, associated with the developed setae (Figure 5B,C). These observations clearly indicate the distal migration of differentiating beta-Oberhautchen cells and also their cornification in the growing free margin.

**Figure 6:**
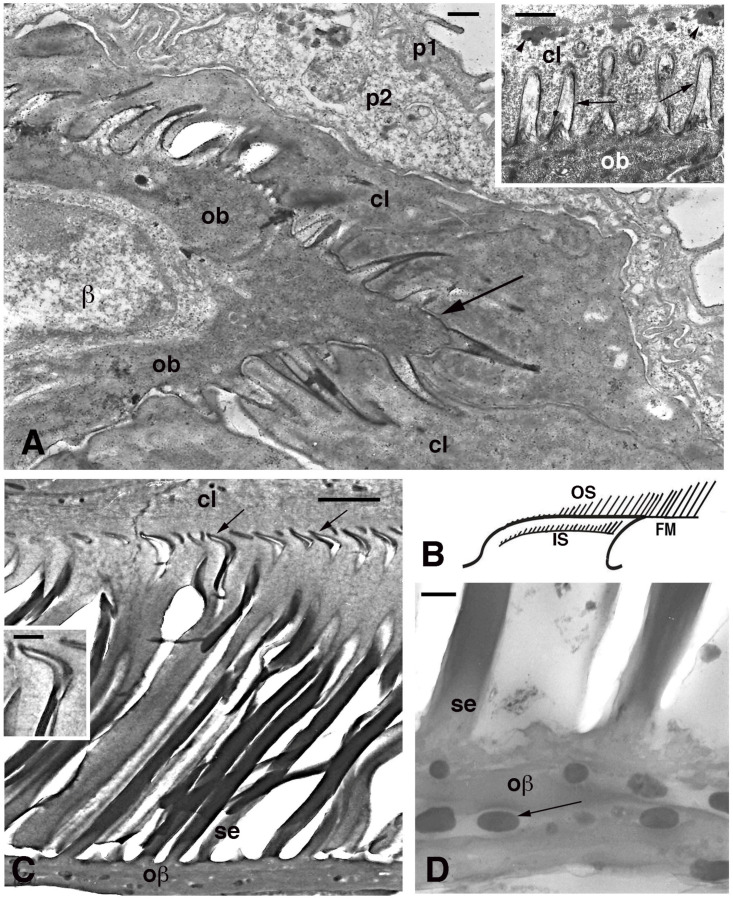
Electron microscopy of seta formation in late embryo of *Anolis lineatopus* (**A**) and adult of *A. carolinensis* (**C**,**D**). (**A**), tip of lamella with apical spinulae forming a rostrum that will produce the free margin (arrow) at a later stage. Bar, 0.5 μm. The inset (Bar, 0.5 μm) shows the initial formation of embryonic setae (arrows) penetrating the clear layer (arrowheads on keratohyalin-like granules). (**B**), schematic drawing illustrating the outer (OS) and inner (IS) setae generations of a mature lamella during the renewal phase. (**C**), detail on inner setae (refers to IS in (**B**)) that are almost mature with their terminal spatulae (arrows). Bar, 1 μm. The inset (Bar, 200 nm) shows the curved shape of the spatula. (**D**), detail on melanosomes (arrows) included within the Oberhautchen-beta-layer of the free margin (refers to FM in (**B**)). Bar, 0.5 μm. **Legend**: β, beta-cell (immature); cl, clear layer; IS, inner setae; ob, Oberhautchen cell; oβ, Oberhautchen merged with the beta-layer; OS, outer setae; p1, outer periderm; p2, inner periderm; se, setae.

**Figure 7:**
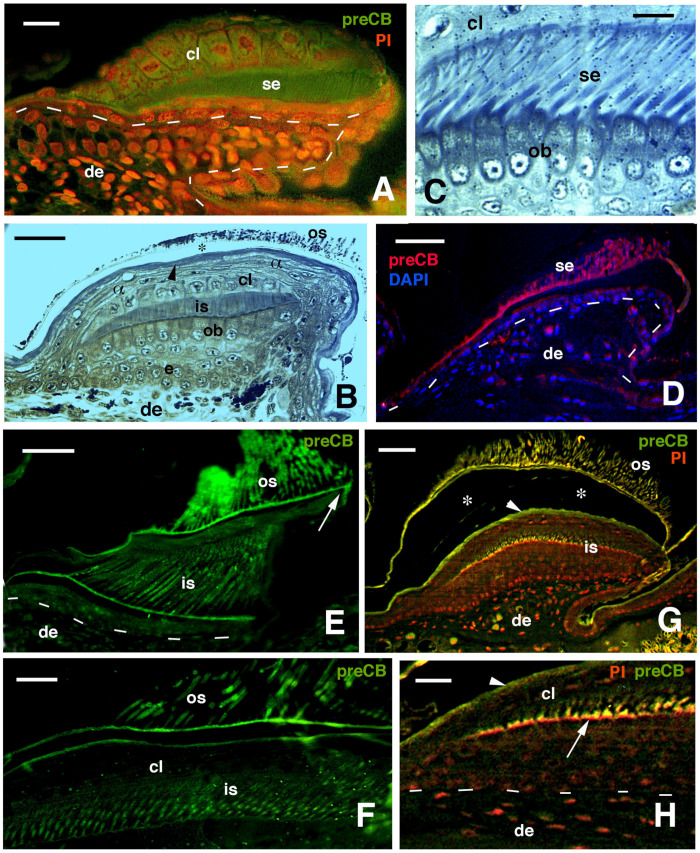
Immunofluorescence for CBPs (corneous beta proteins) using the pre-core box antibody (preCB) on pad lamellae of lizards. (**A**), pad lamella of late embryo of *A. lineatopus* with immunolabeled setae (green) and nuclei fluorescent (red) with propidium iodide (PI). Dashes underline the epidermis. Bar, 10 μm. (**B**), longitudinal and tangential section of lamella of *H. turcicus* showing numerous epidermal layers. The arrowhead indicates the alpha-layer. The asterisk indicates artifact separation (due to sectioning) between outer setae and the alpha-layer. Toluidine blue stain. Bar, 20 μm. (**C**), detail on the shedding complex of *T. mauritanica* with forming setae derived from Oberhautchen cells. Toluidine blue stain. Bar, 10 μm. (**D**), immunolabeled setae (red) and nuclei with DAPI (blue) in lamella of *T. mauritanica*. Dashes underline the epidermis. Bar, 20 μm. (**E**), immunolabeled (green) outer corneous layer and setae and inner corneous layer with its setae in *H. turcicus*. The arrow points to the immunofluorescent free margin. Bar, 20 μm. (**F**), immunolabeled setae and outer corneous layer with setae in *T. mauritanica*. Bar, 10 μm. (**G**), double labeling in outer and early-forming inner setae of *T. mauritanica*. The arrowhead indicates the alpha-layer and the asterisks the artifactual split of the epidermis under sectioning. Bar, 20 μm. (**H**), close-up view of inner setae (arrow) at an early phase of their growth. The arrowhead indicates the alpha-layer. Dashes underline the epidermis. Bar, 10 μm. **Legend**: α, alpha-layer; cl, clear layer; de, dermis; e, epidermis; is, inner setae; ob, Oberhautchen-beta-layer; os, outer setae; se, setae.

The ultrastructural analysis of the free margin in *A. lineatopus* embryos shows that Oberhautchen cells give rise to a rostral corneous and spinous distal elongation that is likely pushed forward by the continuous addition of the migrating Oberhautchen and beta-cells toward the apex of the lamella (Figure 6A,B). The shape of apical Oberhautchen cells appears folded ventrally, toward the inner lamella surface. The setae formed in more proximal areas of the lamellae are electron-pale initially but at maturation their density increases, like in the clear layer that also becomes dense and corneous. Adult lamellae show a very thin (0.3–1.0 μm) beta-layer merged to the Oberhautchen at the base of setae which contain non-corneous areas, possibly still synthetically active, and often occupied with melanosomes (Figure 6C,D). Most of the corneus material of setae and sustaining beta-layer contain corneous beta proteins (CBPs), as indicated by immunolabeling with diverse antibodies against these proteins (Figure 7A,B). In embryos of *Gecko japonicus*, the expression and localization of specific glycine–proline-rich and cysteine–proline-rich CBPs has been detected [27], although a large part of the adult CBPs is also cysteine-rich [4,30,31].

## 3. Renewal of Pad Lamellae in Geckos

During their activity and wandering around their environment, geckos endlessly use their digital pads over different surfaces and for variable periods of time, imposing a lot of stress and potential wear on the setae [3,6]. This requires a frequent replacement of setae in order to maintain their adhesion properties and sticking efficiency, but no specific data on the frequency of digital shedding in comparison to other body regions are available to date. However, it is believed that shedding frequency in digital pads is the same as for other body regions but a specific study on this point, as well as information on the degree of setae wear during prolonged adhesion, is missing. 

**Figure 8:**
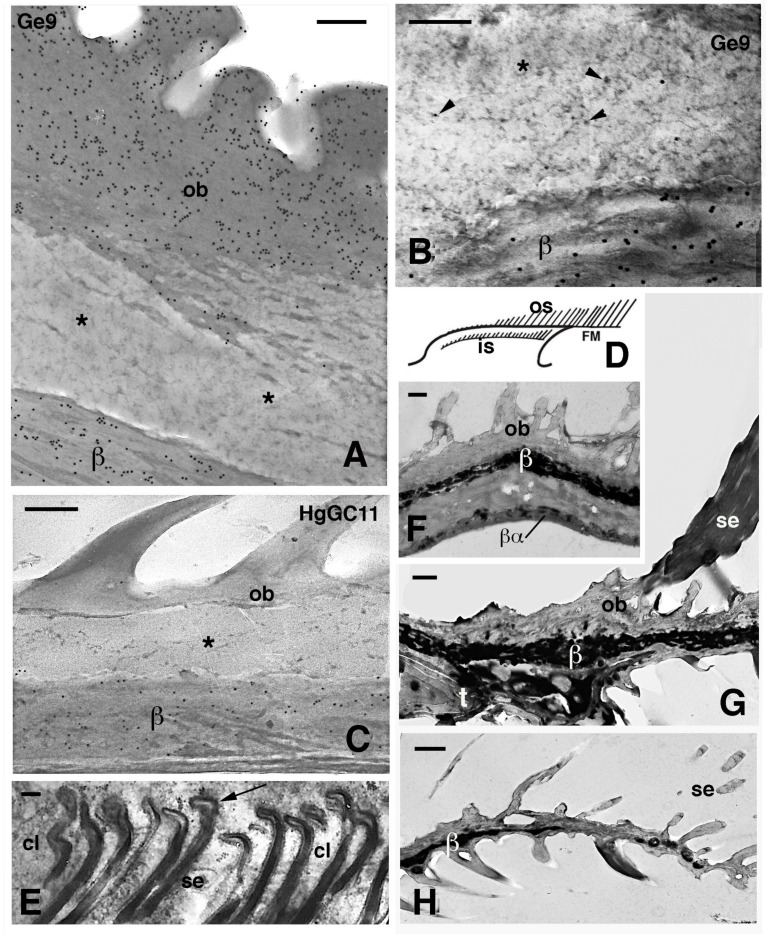
Electron microscopic details of external corneous layers of pad lamellae. (**A**), immunolabeled Oberhautchen and beta-layer in *G. gecko* using the Ge9 antibody for CBPs. Asterisks indicate a pale layer with scarce corneous material sandwiched between the labeled corneous layers. Bar, 200 nm. (**B**), detail of the pale layer (asterisk) containing sparse ribosomes (arrowheads). Ge9 immunolabeling in *G. gecko*. Bar, 100 nm. (**C**), detail of the corneous layer of lamella in *H. turcicus* with the pale layer (asterisk). Immunolabeling using the HgGC11 antibody for CBPs. Bar, 100 nm. (**D**), schematic drawing of lamella evidencing outer and inner setae. (**E**), forming spatula ends (arrows) of the inner setae in *A. carolinensis*. Bar, 200 nm. (**F**), detail of the corneous layer by the apex of the lamella in *G. gecko*. Bar, 0.5 μm. (**G**), detail of the corneous layer at the beginning of the free margin in *G. gecko*. Bar, 0.5 μm. (**H**), tip of the free margin in *G. gecko*. Bar, 0.5 μm. **Legend**: β, beta-layer/corneous material; βα, likely alpha-layer merged with the beta-layer of the lamella (mesos layer absent); cl, clear layer (cell); FM, free margin; is, inner setae; os, outer setae; ob, Oberhautchen; se, setae; t, distal tip of the pad lamella.

The composition of developing and mature setae is likely similar as they are mainly formed by CBPs and a minor component of IF-keratins [4,30,32,33,34]. Moreover, structural lipids are present among the corneous bundles forming the setae [35,36]. The corneous proteins are quite resistant to physical stress but setae are released during shedding with some worn spatulae. The prevalence of resistant CBPs ensures flexibility and endurance of the setae, as most are rich in cysteine and proline while others contain glycine and proline. Immunolabeling analysis with the light and electron microscope have indicated that while the cysteine–proline-rich CBPs form most of the setae, the glycine–proline-rich CBPs constitute the beta-layer at the base of the setae [20,31]. CBPs are maximally packed in the outer setae, as revealed from TEM analysis and from the higher intensity of immunostaining in comparison to the inner setae (Figure 7D–F). These studies have shown that the Oberhautchen and underlying beta-layer are merged in the setae, but in the thicker part of the corneous layer of the lamella, a pale cytoplasmic space still containing sparse ribosomes is present even in the outer setae (Figure 8A–C). This layer, unlabeled using antibodies against CBPs, may act as a form of cushion for the vibrational movements of the overlying outer setae that contact the substrate for adhesion. This pale layer disappears in the thinner corneous layer sustaining the apical setae located in the free margin.

The forming inner setae (Figure 8D) are embedded into the cytoplasm of the clear layer of the outer epidermal generation and complete the formation of new spatulae (Figure 8E). The outer beta-layer and the paler cytoplasm of the apical (distal) part of lamellae thins out and while the pale layer disappears, a thin beta-layer also remains in the free margin and likely sustains its flexibility and maintains the setae upright (Figure 8F–H). The electron microscopic observations therefore indicate that part of the outer corneous layer sustaining the setae may still have some biosynthetic activity.

**Figure 9:**
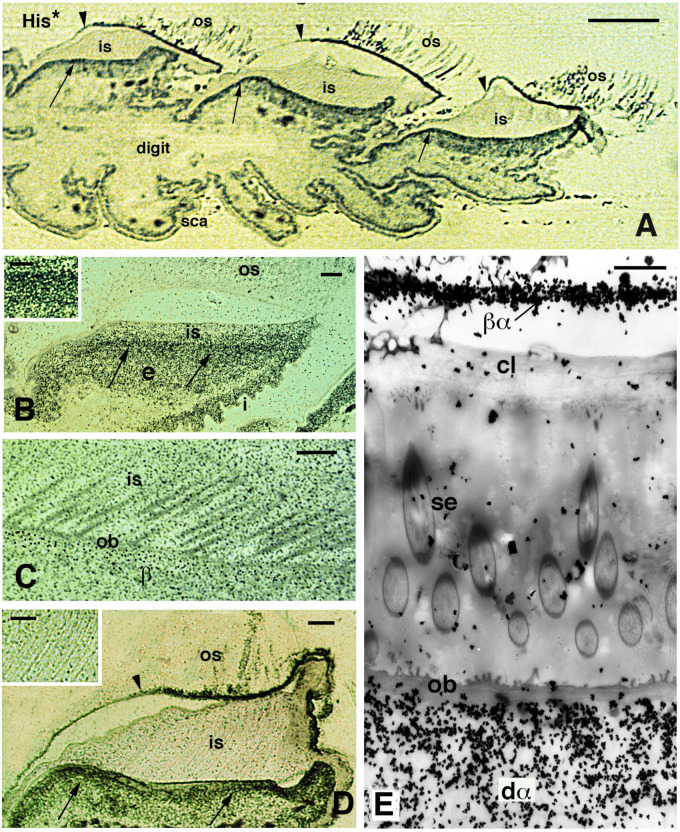
Autoradiographical view of pad lamellae and setae of *H. turcicus* 4 h post-injection of tritiated histidine (His*). (**A**), longitudinal section showing a digit with three pad lamellae (the ventral side is downward). The inner Oberhautchen-beta-layer (arrows) is intensely labeled, while the outer Oberhautchen-beta-layer is mainly labeled in the distal part (indicated by arrowheads). Bar, 100 μm. (**B**), intensely labeled epidermal area occupied by differentiating inner Oberhautchen and beta-cells (arrows). Bar, 10 μm. The inset (Bar, 5 μm) provides detail on the intense labeling of these layers. (**C**), detail of the diffuse autoradiographic labeling in inner setae and Oberhautchen layer in the renewal phase of the lamella epidermis. Bar, 10 μm. (**D**), labeling in the outer Oberhautchen-corneous layer (arrowhead) and inner Oberhautchen-beta-layer (arrows) at a late renewal phase of the epidermis, with inner setae only diffusely labeled. Bar, 10 μm. The inset (Bar, 0.5 μm) shows that the inner setae are almost unlabeled at this stage of the renewal phase. (**E**), electron microscopic autoradiography showing unlabeled inner setae, Oberhautchen-beta-layer and clear layer, while the differentiating inner alpha-cells underneath are heavily labeled. Moreover, numerous silver grains are present over the thin beta-alpha-layer forming the corneous layer sustaining the outer setae. Bar, 1 μm. **Legend**: β, forming beta-layer; βα, corneous layer formed by a thin beta-layer with the alpha-layer; cl, clear layer; dα, differentiating alpha-cells; e, epidermis; i, inner surface of the lamella; is, inner setae; ob, Oberhautchen-beta-layer; os, outer setae; sca, scales; se, setae (inner).

Immunolabeling, amino acid autoradiography, in situ hybridization and TEM observations indicate that since the beginning of their formation, a large number of CBPs accumulates at the base of the forming setae and they mature with the movement inside the setae during their distal growth (Figure 7G,H) [17,20,21,22,32]. Current information indicates that while the inner setae are forming from the Oberhautchen layer of the inner epidermal generation in the lamellae, the long and mature outer setae have moved toward the tip of the lamella (Figure 1F, Figure 2A,B,D and Figure 7D,E). Histological and autoradiographical analyses after tritiated histidine and tritiated proline administration have suggested that the outer setae gradually move apically along the beta-layer during the renewal phase of the epidermis to reach the free margin before being shed (Figure 4B, Figure 7B,C and Figure 9). This hypothesis is suggested by the prevalent labeling along the corneous layer sustaining the outer setae that is formed by a thin beta-layer and alpha-layer. The autoradiographic labeling appears more intense in the distal part of the outer corneous layer present toward the tip of the lamellae, with the labeling low to absent in the proximal part at 4 h post-injection of tritiated proline or histidine (Figure 9A,D). The main sites of localization of the autoradiographic signals vary according to the stage of the renewal phase along the lamellae and the setae. While the outer setae are unlabeled, the inner setae contained the radioactive amino acids with lower intensity than the underlying Oberhautchen and beta-cells (Figure 9B,C), or remain unlabeled like the outer setae (Figure 9A,D,E). Ultrastructural autoradiography suggests that the labeling of the outer corneous layer sustaining the outer setae derive from the incorporation of tritiated amino acids also in the thin alpha-layer associated with the beta-layer of the outer setae, a condition that may further sustain the distal setae movement (Figure 9E). It is known that the full maturation of the outer alpha-layer (lacunar tissue) in numerous species of lizards and snakes occurs when the inner Oberhautchen and beta-layers have already been differentiated underneath [19].

**Figure 10:**
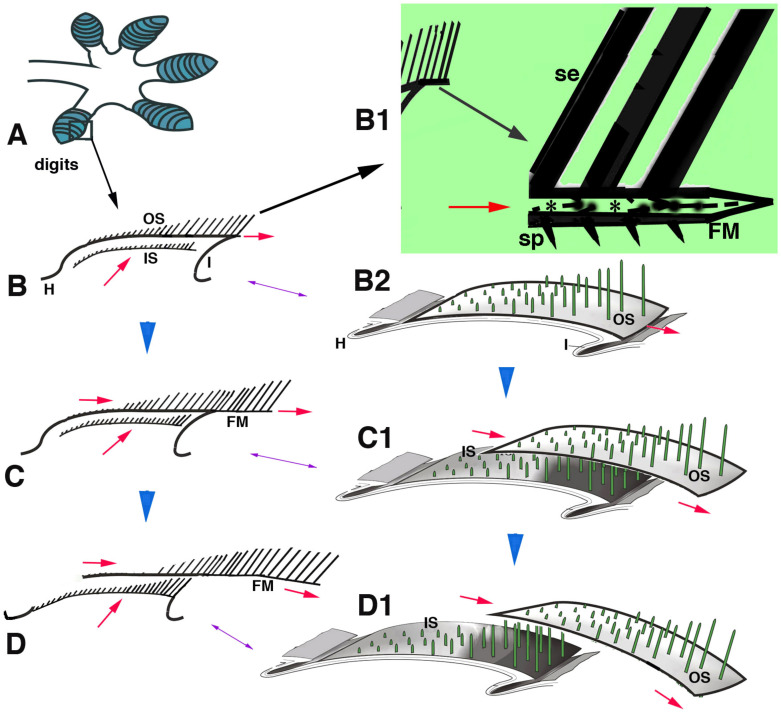
Schematic drawing summarizing the hypothesis of setae replacement in pad lamellae from phase (**B**–**D**) (blue arrowheads). (**A**), digit with lamellae (blue). (**B**), detail of a longitudinally sectioned lamellae with two setae generations. (**B1**), detail of the free margin (large arrow). Asterisks in B1 indicate the pale layer within the Oberhautchen and beta-layer that sustain the setae. (**B2**), three-dimensional representation of a lamella. (**C**), in a following phase, the outer setae move toward the lamella tip, while the inner setae grow underneath (red arrows). (**C1**) shows the distal displacement of the outer setae and the sustaining Oberhautchen-beta-layer. (**D**), in a final phase of shedding, the outer setae are lost from the lamella tip and the inner setae emerge underneath. (**D1**), three-dimensional view of outer setae shedding. **Legend**: FM, free margin; H, hinge region; I, inner lamella surface; IS, inner setae; OS, outer setae; se, setae; sp, spinulae; The red arrows indicate the upper and distal movements of outer and inner setae.

The labeling for histidine and proline is more uniform in the forming setae of the inner generation where no effective distal movement is yet in place (Figure 9B,C). Another indication that the outer beta-layer and associated thin alpha-layer are still metabolically active comes from in situ hybridization studies that showed mRNAs for CBPs still present in this outer layer, but not yet fully cornified [22]. Based on the above studies and on the histological analysis of setae replacement during the shedding cycle [17], we have proposed the model of setae replacement shown in Figure 10. The distal setae are longer and exposed to the substrate while the inner setae are forming underneath (Figure 10A–B2). The apical-most outer setae are moved forward over the free margin (Figure 10B1,C,D) and they gradually detach from the lamella to be shed at maturity of the inner setae (Figure 10C–D1). The free margin is mainly formed by a very flat beta-cell sandwiched by Oberhautchen cells [17,37]. In their hypotheses [17], the researchers indicated that a regression of the dermis of the lamella was more likely than an extension of the outer Oberhautchen-beta-layer to explain the distal shifting of the setae. This may imply that the lamella somehow becomes shorter, except for the free margin. This interpretation would conflict with the fact that scales and pad lamellae are growing and not shrinking [37]. Instead, my hypothesis sustains the contrary, supporting the distal extension of the free margin as pushed distally from the growth of the outer beta- and alpha-corneous layers (Figure 10). Due to the lack of further experiments, the mechanism hypothesized here remains uncertain and should be further experimentally demonstrated. Moreover, the degree of wearing and “stickiness” of the setae after long usage by geckos should be evaluated.

In conclusion, although an abundance of information reported in this review on the ultrastructure, genes, protein composition and formation of setae has been gained in the past 10 years, other questions remain: (a) What determines the length and the specific branching pattern of setae observed in different gecko species? It is known that species-specific patterns of setae derive from the process of growth of the small Oberhautchen spinule into the clear layer, but further information requires specific experimental manipulations, including the in vitro analysis of setae formation. (b) Which cell mechanisms are operating during the growth, distal shifting and final shedding of setae in the pad lamella? (c) Is there a different shedding frequency of the (worn) setae in comparison to other body areas of the skin? (d) What is the specific role of setal proteins in the chemical–physical mechanism of adhesion? The latter study would produce important information for the production of artificial polymers of higher adhesion for multiple applications [31].

## Data Availability

Not applicable.
